# Influence of a Simulated Marine Atmosphere on the Fatigue Performance of TC25 Alloy

**DOI:** 10.3390/ma19122484

**Published:** 2026-06-10

**Authors:** Guangming Kong, Yichen Jiang, Jianglong Ma, Zhiguo Liu, Ang Tian

**Affiliations:** 1 Qingdao Campus, Naval Aviation University, Qingdao 266041, China; 2 School of Metallurgy, Northeastern University, Shenyang 110819, China

**Keywords:** TC25 titanium alloy, marine atmospheric corrosion, high-temperature low-cycle fatigue, hysteresis loop

## Abstract

**Highlights:**

- A systematic investigation of the high-temperature fatigue behavior of TC25 before and after corrosion is reported for the first time.
- Fatigue life prediction models for TC25 titanium alloy before and after corrosion were developed.
- Multiple crack initiation sites induced by corrosion pits are mainly responsible for the reduced fatigue life after corrosion.

**Abstract:**

Titanium alloys have been extensively employed in the aerospace industry, and their service performance is largely governed by high-temperature low-cycle fatigue damage. However, investigations into the fatigue behavior of TC25 titanium alloy subjected to corrosion in a marine atmospheric environment remain limited. In this study, high-temperature low-cycle fatigue tests were conducted on TC25 titanium alloy before and after corrosion. It was found that, after corrosion, the proportion of the structural failure stage increased by approximately 10%. The corrosion pits on the surface led to local stress concentration, resulting in an increase in the number of fatigue crack sources and an acceleration of the fatigue crack growth rate, thus reducing the fatigue life of the material. These findings provide important theoretical and experimental support for the application of TC25 titanium alloy in marine environments.

## 1. Introduction

With the advancement of aviation propulsion technology toward higher thrust-to-weight ratios, higher bypass ratios, and higher turbine inlet temperatures, titanium alloys have been widely used in the aerospace field owing to their high specific strength and excellent corrosion resistance [1,2,3]. With the advancement of aviation propulsion technology, key hot-end components, such as last-stage compressor blades and disks, are subjected to increasingly severe service conditions [4]. As an α+β heat-resistant titanium alloy with an excellent combination of properties [5], TC25 titanium alloy exhibits high specific strength, good heat resistance, and favorable thermal stability at 500–550 °C [6]. Consequently, it has been widely used in critical load-bearing components of advanced aero-engines, such as high-pressure compressor disks, blades, and casings. During service in marine atmospheric environments, such as coastal airports, aircraft engines ingest hot and humid air containing NaCl. The combined action of sodium chloride and water vapor can induce atmospheric corrosion on the surfaces of high-temperature compressor components [7]. After a certain period of service, a corrosion-damaged surface layer develops on the component surfaces. During subsequent flight missions, these components are subjected to low-cycle fatigue loading induced by take-off–cruise–landing cycles [8]. Corrosion significantly alters the surface integrity of the material [9], reduces the fatigue resistance of the alloy, and poses a serious threat to flight safety. Therefore, investigating the high-temperature low-cycle fatigue behavior of corroded titanium alloys is of great importance for ensuring the safety and reliability of aircraft. Extensive research has been conducted on the effects of corrosion on the low-cycle fatigue behavior of metallic materials. Hua et al. [10] investigated the low-cycle fatigue (LCF) behavior of bimetallic steel bar specimens, composed of stainless steel and carbon steel, with varying corrosion ratios. They found that an increased corrosion ratio reduced the fatigue crack propagation area while expanding the final fracture area. Similarly, Chen et al. [11] studied the post-corrosion LCF performance of weathering bridge steel, revealing that corrosion pits on the corroded specimens led to the generation of more crack initiation sites. Furthermore, A. Martin et al. [12] examined the LCF behavior of single-crystal nickel-based superalloys subjected to hot-salt corrosion. Their results demonstrated that pre-corrosion degraded the fatigue resistance due to a higher density of crack initiation points. However, the post-corrosion LCF behavior of titanium alloys, particularly TC25 alloy, remains insufficiently investigated.

When materials are exposed to marine atmospheric environments for prolonged periods, marine atmospheric corrosion readily occurs [13]. Compared with industrial atmospheres, marine atmospheres are generally more corrosive to metallic materials [14]. Consequently, extensive research has been conducted on the corrosion behavior of materials in marine atmospheric environments. Li et al. [15] investigated the fatigue behavior of 30CrMnSiA high-strength steel after marine atmospheric corrosion and found that corrosion transformed the crack initiation mode during fatigue fracture from single-site initiation to multiple-site initiation while also increasing the number of secondary cracks, thereby reducing fatigue life. Song et al. [16] studied additively manufactured AlSi10Mg alloy and reported that pre-corrosion reduced the fatigue life of the alloy by 18–37%. In the pre-corroded specimens, cracks were initiated by edge-valley-shaped corrosion features or by their combined effect with internal defects, and the fracture surfaces exhibited quasi-cleavage characteristics. In addition, pre-corrosion altered the crack initiation location and propagation path. Li et al. [17] investigated 2A70-T6 aluminum alloy and found that, in marine atmospheric environments, the alloy mainly underwent pitting corrosion and intergranular corrosion, which led to multiple crack initiation and a 50% reduction in fatigue life.

However, current studies on titanium alloys have mainly focused on hot-salt corrosion during high-temperature service and its influence on fatigue behavior [18,19,20]. Extensive experimental evidence indicates that, in salt-rich environments above 300 °C, the fatigue limit of titanium alloys decreases significantly, and the fatigue life of some alloys may even be reduced by more than one order of magnitude [21,22]. Shi et al. [23] investigated the hot-salt corrosion fatigue behavior of TC11 titanium alloy after pre-hot-salt-corrosion treatment at 500 °C and found that pre-hot-salt corrosion reduced the fatigue resistance of the alloy by aggravating surface damage, accelerating hydrogen ingress, and promoting hydride formation. When the NaCl deposition amount was greater than or equal to 1 mg/cm^2^, the susceptibility of TC11 alloy to high-temperature stress corrosion cracking increased significantly. Nevertheless, titanium alloys are also susceptible to marine atmospheric corrosion in marine environments. Most previous studies have focused on the corrosion products formed after exposure and the corrosion mechanisms involved [24]. Zhao et al. [25] investigated the corrosion behavior of TC4 titanium alloy in inland and coastal industrial environments and showed that high concentrations of aggressive ions, such as sulfate and chloride ions, together with high relative humidity in marine atmospheres, significantly intensified electrochemical corrosion, resulting in a marked deterioration in the tensile properties of the material.

However, studies on the high-temperature low-cycle fatigue behavior of titanium alloys after accelerated pre-corrosion in a marine atmospheric environment remain limited, and the mechanisms governing the fatigue fracture process are still not fully understood. In this study, TC25 titanium alloy was subjected to 10 cycles of accelerated pre-corrosion in a marine atmospheric environment, and high-temperature low-cycle fatigue tests were performed on the specimens before and after corrosion. The effects of accelerated marine atmospheric pre-corrosion on the fatigue properties of TC25 titanium alloy, as well as the underlying mechanisms, were systematically investigated.

## 2. Materials and Methods

### 2.1. Materials

Our previous study showed that heat treatment improved the mechanical properties of TC25 titanium alloy [26]. Accordingly, TC25 titanium alloy used in the present study was heat-treated as follows: solution treatment at 960 °C (below Tβ) for 2 h, followed by air cooling to room temperature, and then aging at 550 °C for 3 h, followed by air cooling to room temperature. Argon was used as the protective atmosphere during both the solution and aging treatments. The main chemical composition of the alloy is listed in Table 1.

### 2.2. Accelerated Corrosion Testing

**Figure 1:**
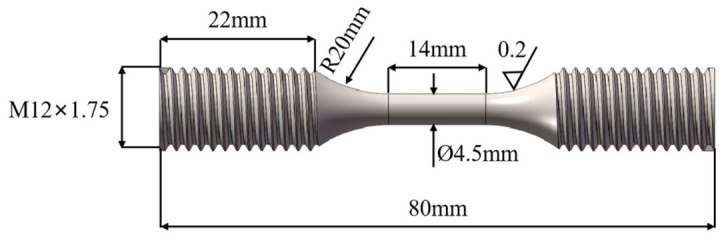
Geometry and dimensions of the low-cycle fatigue specimen (in mm).

**Figure 2:**
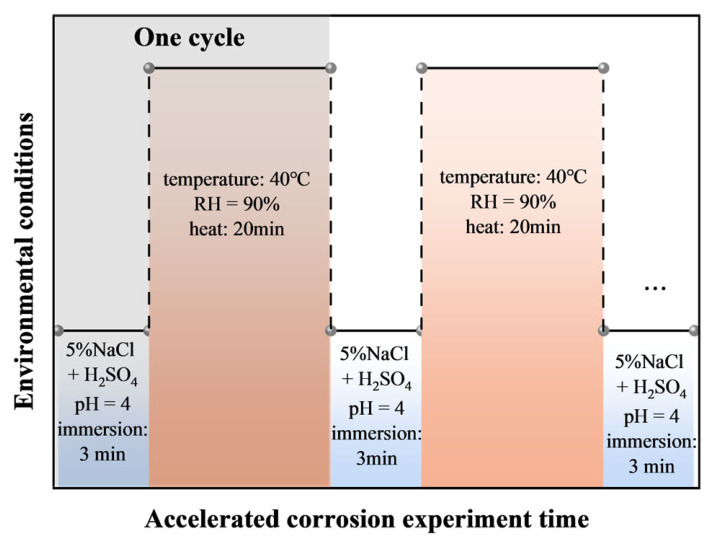
Schematic illustration of the accelerated corrosion cycle.

The heat-treated TC25 titanium alloy was machined into specimens for accelerated corrosion testing, as shown in Figure 1 (the specimens were polished to a surface roughness of Ra = 0.2 μm). The tests were performed in a cyclic immersion corrosion chamber (AISRY, KLD-1200L, Dongguan, China). The accelerated test parameters were designed according to the relevant standard [27] and the actual marine service conditions of titanium alloy power components. Furthermore, the laboratory results were correlated with field-exposed specimens based on the equivalent damage principle ($Q_{t}=Q_{w}$, where $Q_{t}$ and $Q_{w}$ denote the corrosion damage under experimental and actual service conditions, respectively) [28,29]. Therefore, calculations indicate that 128.4 h of accelerated testing in a 5 wt.% NaCl solution at pH 4, an air temperature of 40 °C, and 90% relative humidity is intended to provide a reasonable approximation of one year of actual marine atmospheric exposure. By collecting climatic data from the marine environment under study, it was determined that the average number of wet–dry cycles in the past five years was 335 [30], with a wet-to-dry time ratio of 3:20. Accordingly, an accelerated environmental spectrum was designed with the following parameters: a total cycle duration of 23 min, including 3 min of immersion and 20 min of drying. The environmental conditions, chamber humidity, and accelerated corrosion scheme are shown in Figure 2. Each accelerated corrosion cycle consisted of two stages: immersion in the corrosive solution for 3 min and drying under infrared irradiation for 20 min. A total of 335 cycles constituted one period, corresponding to one year of marine atmospheric corrosion exposure. In total, 10 periods were performed to simulate 10 years of marine atmospheric corrosion.

However, accelerated corrosion testing has certain limitations. Although standardized cyclic immersion helps evaluate the extent of corrosion under controlled parameters, it primarily focuses on the wet–dry alternations and infrared irradiation of a specific marine atmospheric environment and cannot fully replicate the complex, dynamic synergistic effects present in reality. Variables such as microbiologically influenced corrosion and non-steady meteorological alternations during actual service could alter the long-term corrosion behavior of TC25 alloy, which cannot be completely captured by the simulated environmental spectrum. The results of this experiment are applicable as a baseline reference for the studied marine region rather than a universal standard applicable to all different marine climates.

### 2.3. Low-Cycle Fatigue Testing

**Figure 3:**
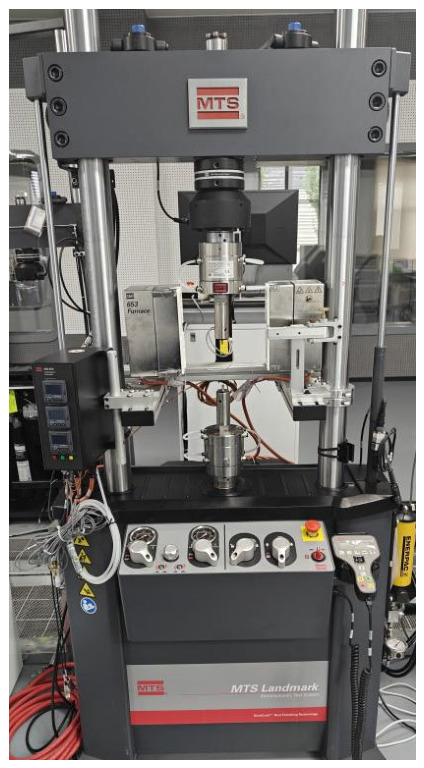
Photograph of the MTS fatigue testing system.

Isothermal uniaxial low-cycle fatigue (LCF) tests were conducted in accordance with the national standard GB/T 15248 [31] (equivalent to ISO 12106 [32]) using an MTS fatigue testing system (Figure 3). The specimens were machined into a round-bar geometry, and the detailed dimensions are shown in Figure 1. Before testing, the gauge-section dimensions were precisely measured using a tool microscope with a precision of 0.001mm to ensure dimensional accuracy. The tests were carried out under total strain control with a triangular waveform. The temperature was maintained at 500 °C, with a strain ratio of R=−1 and a constant strain rate of $3\times 10^{-3}\;s^{-1}$. The test was terminated when either the specimen fractured completely or the cyclic peak load (stress) dropped by 20% relative to the stabilized value. The fatigue life, $N_{f}$, at each stress level was determined based on a 50% survival probability and a 90% confidence level.

To clarify the differences in the high-temperature low-cycle fatigue behavior of TC25 titanium alloy before and after corrosion, a series of microstructural and fracture-surface observations were performed. The microstructure was characterized using a scanning electron microscope (SEM, JEOL JSM-7800F, Tokyo, Japan) operated at an accelerating voltage of 15 kV in LDF mode. Before observation, the fatigue fracture surfaces were cleaned with alcohol and then examined by SEM.

## 3. Results and Discussion

### 3.1. Cyclic Stress Response

Hysteresis loops and stress–amplitude–life curves are key tools for characterizing the cyclic stress response behavior of low-cycle fatigue (LCF). Hysteresis loops not only reflect the evolution of plastic deformation energy [33], but also enable the extraction of key parameters, such as plastic strain amplitude, back stress, and friction stress [34,35]. Stress–amplitude–life curves, in turn, directly reveal the cyclic hardening/softening characteristics of the material. These response features provide an important basis for interpreting the microscopic deformation mechanisms and fatigue life evolution during the LCF process.

#### 3.1.1. Hysteresis Loops

**Figure 4:**
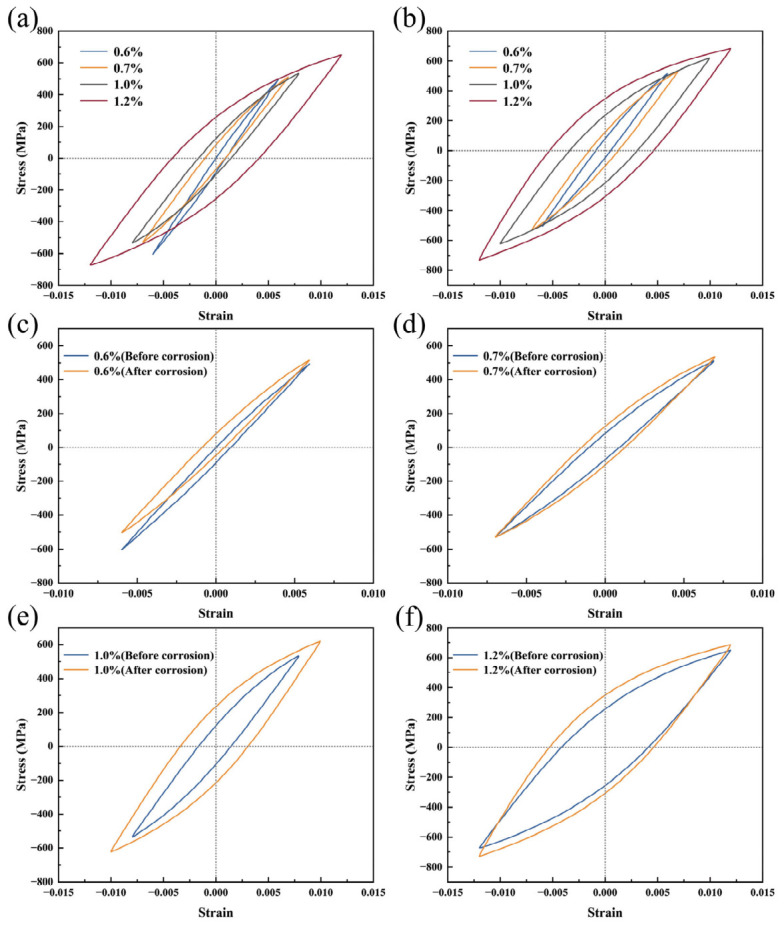
Mid-cycle hysteresis loops obtained from LCF tests at different strain amplitudes before and after corrosion: (**a**) hysteresis loops at all strain amplitudes before corrosion; (**b**) hysteresis loops at all strain amplitudes after corrosion; (**c**) comparison of hysteresis loops before and after corrosion at a strain amplitude of 0.6%; (**d**) comparison of hysteresis loops before and after corrosion at a strain amplitude of 0.7%; (**e**) comparison of hysteresis loops before and after corrosion at a strain amplitude of 1.0%; and (**f**) comparison of hysteresis loops before and after corrosion at a strain amplitude of 1.2%.

Figure 4a,b show the mid-cycle hysteresis loops obtained from the LCF tests at different strain amplitudes before and after corrosion, respectively. The applied strain amplitudes before and after corrosion were 0.6%, 0.7%, 1.0%, and 1.2%. Figure 4c–f compare the mid-cycle hysteresis loops before and after corrosion at strain amplitudes of 0.6%, 0.7%, 1.0%, and 1.2%, respectively. The area enclosed by the hysteresis loop represents the plastic strain energy density dissipated by the material during a single cycle. According to Morrow’s energy dissipation theory [36,37], this energy serves as the driving force for fatigue damage accumulation. It can be seen that both before and after corrosion, the width of the hysteresis loop increases with increasing strain amplitude, whereas the stress level decreases. As the strain amplitude increases and plastic deformation becomes more pronounced, the area enclosed by the hysteresis loop gradually increases, indicating that the material absorbs more energy at higher strain amplitudes. Compared with the uncorroded condition, the area of the mid-cycle hysteresis loop after corrosion is larger, indicating that corrosion leads to greater plastic energy dissipation and damage during the LCF process and suggesting a reduction in the material’s resistance to plastic deformation. This intensification of irreversible energy dissipation implies an accelerated accumulation of internal micro-damage, thereby reducing the resistance to fatigue crack initiation [38] and propagation and ultimately leading to a shorter fatigue life.

#### 3.1.2. Cyclic Stress Response Curves

**Figure 5:**
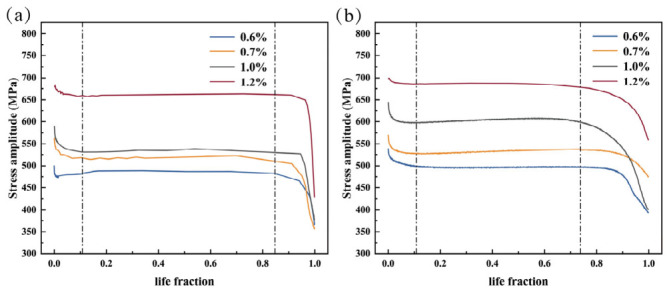
Cyclic stress response curves before and after corrosion: (**a**) before corrosion; (**b**) after corrosion.

The cyclic stress response curves of TC25 titanium alloy during low-cycle fatigue tests before and after corrosion are shown in Figure 5. Figure 5a,b present the relationships between cyclic stress amplitude and life fraction (*n*/*N*_f_; *n* is the actual number of cycles, and *N*_f_ is the total fatigue life) before and after corrosion, respectively. The cyclic stress response can be divided into three main stages [39]. The first stage is the initial rapid softening stage, during which the stress amplitude decreases sharply. This is followed by the progressive stable softening stage, which accounts for the majority of the fatigue life. The final stage is the unstable fracture stage, in which the stress amplitude drops steeply with the rapid propagation of macroscopic cracks until specimen failure occurs. As shown in Figure 5a, before corrosion, the progressive stable softening stage accounts for approximately 75% of the total fatigue life, the unstable fracture stage accounts for approximately 15%, and the initial rapid softening stage accounts for approximately 10%. By contrast, as shown in Figure 5b, after corrosion, the progressive stable softening stage accounts for approximately 65% of the total fatigue life, the unstable fracture stage accounts for approximately 25%, and the initial rapid softening stage still accounts for approximately 10%.

#### 3.1.3. Softening Degree

To quantitatively characterize the evolution of stress amplitude during low-cycle fatigue, the cyclic softening parameter [38] is defined as *η*.

(1) $$\eta =\frac{\sigma_{\mathrm{ini}\;}-\sigma_{i}}{\sigma_{\mathrm{ini}}}$$

**Figure 6:**
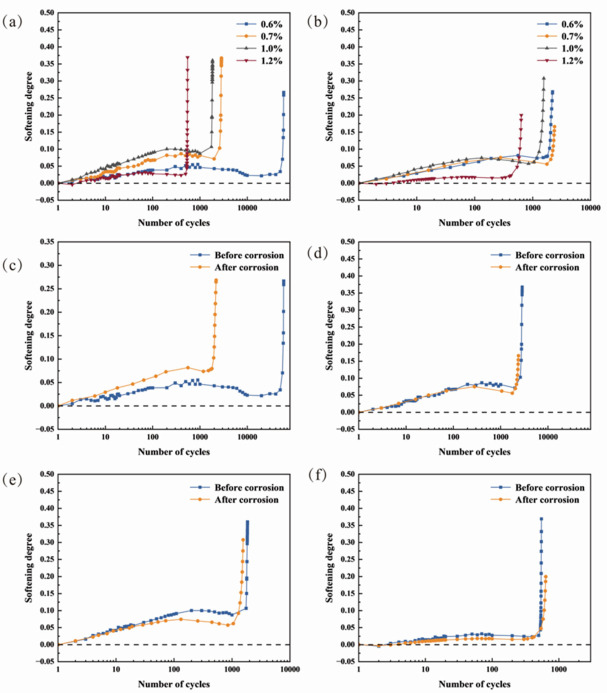
Cycle number–softening parameter curves: (**a**) cycle number–softening parameter curve before corrosion; (**b**) cycle number–softening parameter curve after corrosion; (**c**) comparison of cycle number–softening parameter curves before and after corrosion at a strain amplitude of 0.6%; (**d**) comparison of cycle number–softening parameter curves before and after corrosion at a strain amplitude of 0.7%; (**e**) comparison of cycle number–softening parameter curves before and after corrosion at a strain amplitude of 1.0%; and (**f**) comparison of cycle number–softening parameter curves before and after corrosion at a strain amplitude of 1.2%.

Here, *η* denotes the softening parameter, *σ*_ini_ is the initial stress amplitude, and *σ*_i_ is the stress amplitude at the i~th cycle (i = 2, 3, 4…). It should be noted that *η* < 0 indicates cyclic softening, whereas *η* > 0 indicates cyclic hardening. The relationships between cycle number and softening parameter are shown in Figure 6. Figure 6a,b present the cycle number–softening parameter curves before and after corrosion, respectively. Figure 6c–f compare the softening parameter before and after corrosion at strain amplitudes of 0.6%, 0.7%, 1.0%, and 1.2%, respectively. The results indicate that TC25 alloy generally exhibits a softening trend both before and after corrosion. At a strain amplitude of 1.2%, the alloy shows an initial hardening trend followed by softening, although the extent of hardening is relatively limited. After corrosion, compared with the uncorroded condition, the softening rate increases significantly at a strain amplitude of 0.6%. At strain amplitudes of 0.7%, 1.0%, and 1.2%, however, the softening behavior changes only slightly before and after corrosion.

#### 3.1.4. Back Stress and Friction Stress

**Figure 7:**
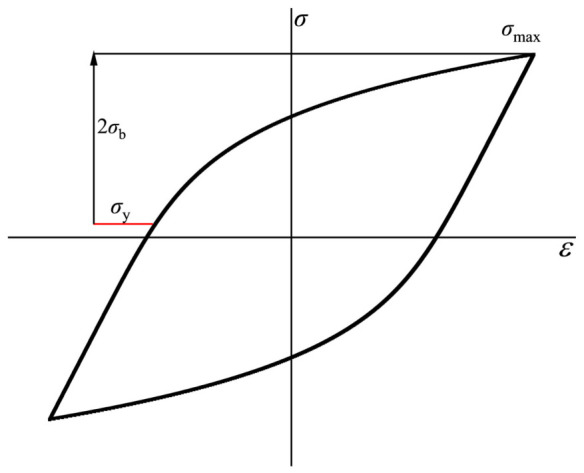
Schematic illustration of the extraction of back stress and friction stress from the hysteresis loop.

To deeply interpret the macroscopic cyclic hardening and softening behaviors of materials during low-cycle fatigue (LCF), micro-mechanical analysis can be carried out by decoupling the evolution laws of back stress ($\sigma_{b}$) and frictional stress ($\sigma_{f}$). From a materials science perspective, $\sigma_{b}$ represents the long-range interaction between mobile dislocations [40] and internal microstructural obstacles [41] in the material, and its magnitude directly reflects the deformation inhomogeneity and the degree of plastic strain incompatibility within the material. In contrast, $\sigma_{f}$ represents the local internal stress required to overcome short-range resistance during dislocation slip, which is mainly controlled by lattice friction and short-range obstacles such as fine precipitates [42,43]. Referring to the classical method proposed by Kuhlmann-Wilsdorf et al. [44,45], this study quantitatively extracted $\sigma_{b}$ and $\sigma_{f}$ from the stress–strain hysteresis loops under different strain amplitudes. Considering the symmetry of the hysteresis loop, this work mainly calculated the parameters based on the tensile loading branch, as shown in Figure 7 [46]. The inflection point on the compression curve, where the transition from the linear elastic stage to the macroscopic plastic deformation stage occurs, is defined as the compressive yield stress $\sigma_{y}$. Subsequently, the values of $\sigma_{b}$ and $\sigma_{f}$ can be calculated through Formulas (2) and (3):

(2) $$\sigma_{f}\;=\;\left(\sigma_{\max}\;-\;\sigma_{y}\right)/2$$

(3) $$\sigma_{b}=\sigma_{\max}\;-\;\sigma_{f}$$

**Figure 8:**
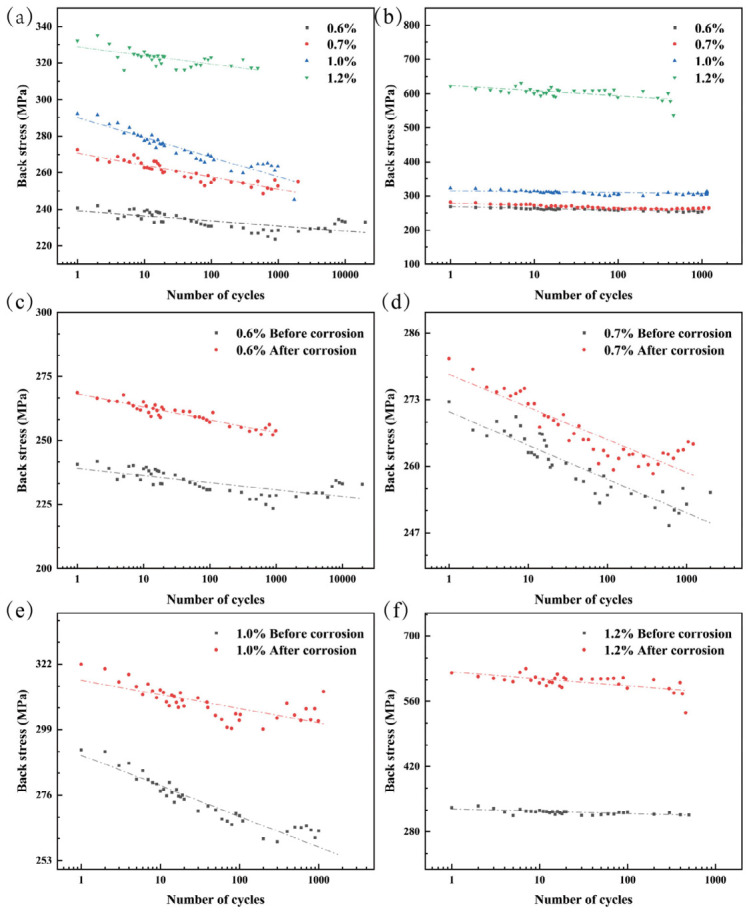
Evolution of back stress with cycle number: (**a**) evolution of back stress with cycle number at different strain amplitudes before corrosion; (**b**) evolution of back stress with cycle number at different strain amplitudes after corrosion; (**c**) comparison of back-stress evolution with cycle number before and after corrosion at a strain amplitude of 0.6%; (**d**) comparison of back-stress evolution with cycle number before and after corrosion at a strain amplitude of 0.7%; (**e**) comparison of back-stress evolution with cycle number before and after corrosion at a strain amplitude of 1.0%; and (**f**) comparison of back-stress evolution with cycle number before and after corrosion at a strain amplitude of 1.2%.

Figure 8 shows the evolution of back stress ($\sigma_{b}$) with cycle number before and after corrosion. Figure 8a presents the evolution of $\sigma_{b}$ with cycle number at different strain amplitudes before corrosion, illustrating the back-stress evolution in uncorroded specimens at strain amplitudes of 0.6%, 0.7%, 1.0%, and 1.2%. At all tested strain amplitudes, the back stress gradually decreased with increasing cycle number. Meanwhile, the back-stress level exhibited a pronounced dependence on strain amplitude, with the initial back stress increasing progressively as the strain amplitude increased. As the strain amplitude increased from 0.6% to 1.2%, the initial back stress increased from approximately 240 MPa to approximately 330 MPa. Figure 8b shows the evolution of $\sigma_{b}$ with cycle number at different strain amplitudes after corrosion. Similar to the uncorroded condition, the back stress at each strain amplitude generally showed a decreasing trend during cycling. Among them, the back-stress values at strain amplitudes of 0.6%, 0.7%, and 1.0% were relatively close, mainly ranging from 250 to 320 MPa. By contrast, at the high strain amplitude of 1.2%, the back stress increased markedly to above 600 MPa, which was substantially higher than those at the lower strain amplitudes. Figure 8c–f further compare the evolution of back stress before and after corrosion at strain amplitudes of 0.6%, 0.7%, 1.0%, and 1.2%, respectively. At a strain amplitude of 0.6% (Figure 8c), the back-stress–cycle number curve of the corroded specimen shifted upward as a whole and remained approximately 25–30 MPa higher than that of the uncorroded specimen throughout the entire fatigue life, while both decreased at nearly the same rate with increasing cycle number. At a strain amplitude of 0.7% (Figure 8d), the initial back stress of the corroded specimen was slightly higher than that of the uncorroded specimen. As cycling proceeded, however, the back stress of the corroded specimen decreased more rapidly, and when the cycle number reached approximately 1000, the back-stress values before and after corrosion gradually converged. At a strain amplitude of 1.0% (Figure 8e), the back-stress level after corrosion increased significantly. Throughout the entire cycling process, the back stress of the corroded specimen remained markedly higher than that of the uncorroded specimen. Meanwhile, the back stress of the uncorroded specimen decreased slightly faster than that of the corroded specimen. At a strain amplitude of 1.2% (Figure 8f), the increase in back stress after pre-corrosion was most pronounced. The back stress of the uncorroded specimen remained around 320 MPa and exhibited a slight downward trend, whereas that of the corroded specimen increased sharply to above 600 MPa and then decreased gradually while remaining at an extremely high level throughout the entire cycling process.

**Figure 9:**
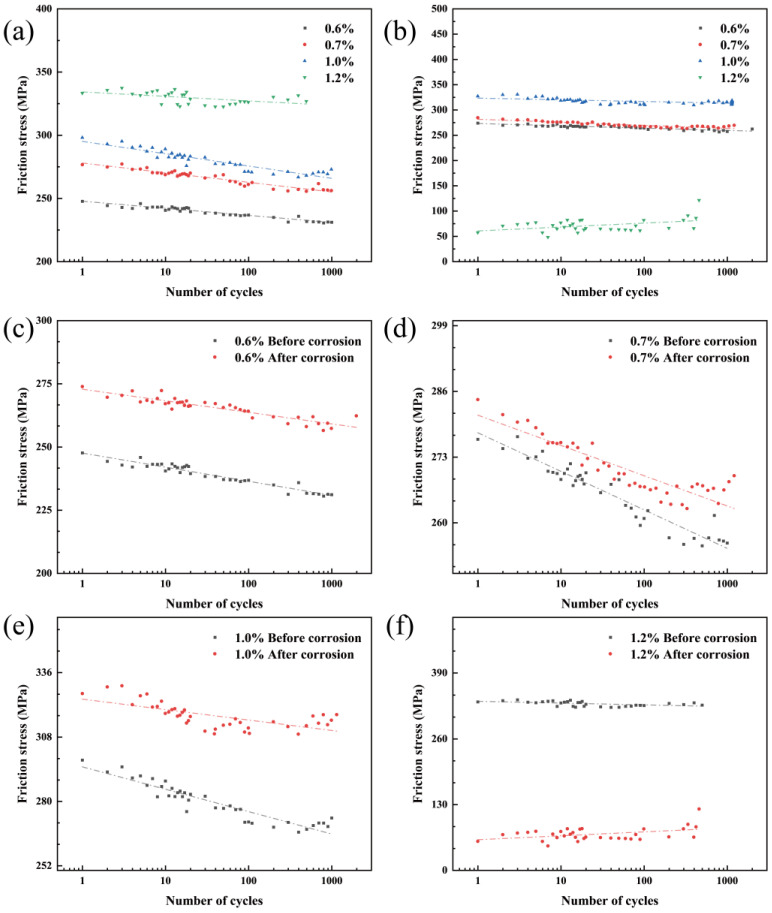
Evolution of friction stress with cycle number: (**a**) evolution of friction stress with cycle number at different strain amplitudes before corrosion; (**b**) evolution of friction stress with cycle number at different strain amplitudes after corrosion; (**c**) comparison of friction–stress evolution with cycle number before and after corrosion at a strain amplitude of 0.6%; (**d**) comparison of friction–stress evolution with cycle number before and after corrosion at a strain amplitude of 0.7%; (**e**) comparison of friction–stress evolution with cycle number before and after corrosion at a strain amplitude of 1.0%; and (**f**) comparison of friction–stress evolution with cycle number before and after corrosion at a strain amplitude of 1.2%.

Figure 9a shows the evolution of friction stress with cycle number in uncorroded specimens at different strain amplitudes (0.6%, 0.7%, 1.0%, and 1.2%). Within the tested strain-amplitude range, the friction stress gradually decreased with increasing cycle number. In addition, the friction–stress level increased significantly with increasing strain amplitude. As the strain amplitude increased from 0.6% to 1.2%, the friction stress increased progressively, and its value range rose from approximately 220–250 MPa to 310–340 MPa. Figure 9b presents the evolution of friction stress in specimens after pre-corrosion. At strain amplitudes of 0.6%, 0.7%, and 1.0%, the friction stress still showed a decreasing trend with increasing cycle number, and the values were mainly distributed in the range of 250–330 MPa. However, at the high strain amplitude of 1.2%, the friction stress decreased sharply to 50–120 MPa and then exhibited a slight upward trend during subsequent cycling. At a strain amplitude of 0.6% (Figure 9c), the friction stress of the corroded specimen remained higher than that of the uncorroded specimen throughout the entire fatigue life, with a difference of approximately 20–25 MPa. In both cases, the friction stress decreased slowly with increasing cycle number, and the slopes of the two curves were nearly parallel. At a strain amplitude of 0.7% (Figure 9d), the friction stress before and after corrosion both exhibited a relatively obvious downward trend, while the friction stress of the corroded specimen remained slightly higher overall. At a strain amplitude of 1.0% (Figure 9e), the friction–stress level increased significantly after pre-corrosion. The friction stress of the uncorroded specimen decreased relatively rapidly with increasing cycle number, whereas that of the corroded specimen, although having a higher initial value (approximately 320 MPa), showed a more gradual downward trend. At a strain amplitude of 1.2% (Figure 9f), the evolution of friction stress before and after corrosion differed completely. The friction stress of the uncorroded specimen remained relatively stable at a high level of approximately 330 MPa, whereas that of the corroded specimen decreased significantly, remained below 100 MPa, and exhibited a slight upward trend as cycling proceeded.

Combined with the evolution of the softening parameter (Figure 6), the response characteristics of the internal stresses ($\sigma_{b}$ and $\sigma_{f}$) indicate that, during the initial stage of cyclic deformation in TC25 titanium alloy, the rapid increase in the softening parameter corresponds to the rapid decay of $\sigma_{b}$ and $\sigma_{f}$ in the early cycles, reflecting a rapid reduction in the initial resistance of the material to plastic deformation. Subsequently, the alloy enters a progressive stable softening stage, which occupies most of the fatigue life. In this stage, the alloy exhibits continuous softening, and the softening–parameter curve shows an approximately linear upward trend. This behavior is consistent with the continuous decrease in $\sigma_{b}$ and $\sigma_{f}$, indicating that damage evolution and microstructural softening persist throughout the fatigue process. In the final unstable fracture stage, the sudden drop in the apparent stress signifies the rapid propagation of macroscopic cracks. Further comparison shows that pre-corrosion modifies the softening behavior of the material, and this effect is strongly strain-amplitude dependent. At low strain amplitudes, corrosion damage promotes overall softening. In contrast, at a high strain amplitude of 1.2%, the coupled effect of high-strain loading and pre-corrosion-induced surface defects causes a sharp increase in back stress (approximately 600 MPa), thereby driving TC25 titanium alloy into the fracture stage prematurely. Overall, the two internal stresses in TC25 titanium alloy exhibit completely opposite variation trends. On the one hand, corrosion damages the local microstructure, acting as an additional short-range barrier at low strains that increases the friction stress, whereas at high strains, corrosion defects lead to severe stress concentration, dismantling the short-range barriers on the slip bands, significantly reducing the resistance to dislocation slip (with the friction stress dropping below 100 MPa) and making local regions highly susceptible to deformation [47]. On the other hand, the surface inhomogeneity caused by corrosion pits forces massive dislocations to pile up at the defects, leading to a rapid increase in long-range resistance (with the back stress surging to about 600 MPa). It is precisely the superposition of this sudden drop in friction stress and the drastic increase in back stress that directly accelerates micro-crack generation, leading to the early fracture of the pre-corroded specimens [23].

### 3.2. Fatigue Life and Fractographic Analysis

Under strain-controlled low-cycle fatigue conditions, the total strain amplitude ($\frac{∆\varepsilon }{2}$) is composed of the elastic strain amplitude ($\frac{∆\varepsilon_{e}}{2}$) and the plastic strain amplitude ($\frac{∆\varepsilon_{p}}{2}$). To accurately characterize the fatigue life behavior of TC25 titanium alloy, the Basquin–Coffin–Manson (B-C-M) relationship was adopted to describe the correlation between total strain amplitude and fatigue life ($N_{f}$). This model combines the Basquin elastic strain–life law with the Coffin–Manson plastic strain–life law [48], and is expressed as follows:

(4) $$\frac{∆\varepsilon }{2}=\frac{∆\varepsilon_{e}}{2}+\frac{∆\varepsilon_{p}}{2}=\frac{{\sigma^{\prime }}_{f}}{E^{*}}{{(2N}_{f})}^{b}+{\varepsilon^{\prime }}_{f}{{(2N}_{f})}^{c}$$

where $\frac{∆\varepsilon }{2}$ is the total strain amplitude; ${2N}_{f}$ is the number of reversals to failure; $E^{*}$ is the cyclic elastic modulus; ${\sigma^{\prime }}_{f}$ is the fatigue strength coefficient; b is the fatigue strength exponent; ${\varepsilon^{\prime }}_{f}$ is the fatigue ductility coefficient; and c is the fatigue ductility exponent.

The fatigue life equation before corrosion is as follows:

(5) $$\frac{∆\varepsilon }{2}=0.01122{\left({2N}_{f}\right)}^{-0.06277}+0.86922{\left({2N}_{f}\right)}^{-0.72943}$$

The fatigue life equation after corrosion is as follows:

(6) $$\frac{∆\varepsilon }{2}\;=\;0.02027{\left({2N}_{f}\right)}^{0.14725}\;+\;0.7121{\left({2N}_{f}\right)}^{-0.71782}$$

**Figure 10:**
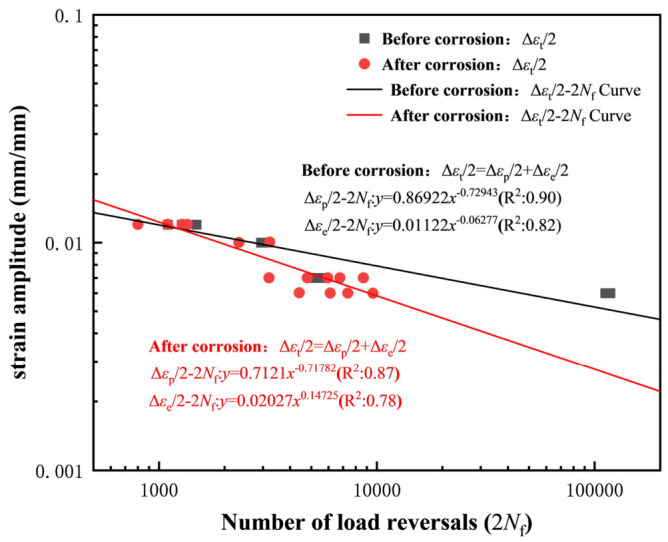
Comparison of the high-temperature low-cycle fatigue life curves of TC25 titanium alloy before and after corrosion.

Figure 10 presents a comparison of the high-temperature low-cycle fatigue life curves of TC25 titanium alloy before and after corrosion. Although the fatigue life of TC25 titanium alloy falls within the conventional low-cycle fatigue regime, the strain decomposition results indicate that elastic strain dominates the total strain in the present study, accounting for approximately 50–90% of the total strain. This suggests that the fatigue behavior of the alloy is not characteristic of typical plasticity-dominated low-cycle fatigue but instead exhibits pronounced features of the transition regime between low-cycle fatigue and high-cycle fatigue [49]. As shown in Figure 10, the strain–life curve of TC25 titanium alloy shifts downward after corrosion, indicating a significant reduction in fatigue life under the same total strain amplitude. This demonstrates that corrosion markedly deteriorates the high-temperature fatigue performance of the alloy. Corrosion affects both the crack initiation and crack propagation stages, thereby influencing the damage processes described by the Basquin and Coffin–Manson terms and ultimately reducing the overall fatigue life. To further elucidate the effect of corrosion on the fatigue behavior of TC25 titanium alloy, the Basquin–Coffin–Manson model parameters before and after corrosion were compared. The results show that the fatigue ductility coefficient decreases significantly after corrosion, whereas the fatigue ductility exponent changes only slightly. This indicates that corrosion does not alter the fundamental evolution law of fatigue-related plastic damage but significantly weakens the plastic deformation capacity of the material, thereby promoting crack initiation [17]. Meanwhile, the decrease in the fatigue strength coefficient indicates a reduction in the load-bearing capacity of the material, whereas the variation in the fatigue strength exponent suggests a change in the sensitivity of the material to stress level [50].

**Figure 11:**
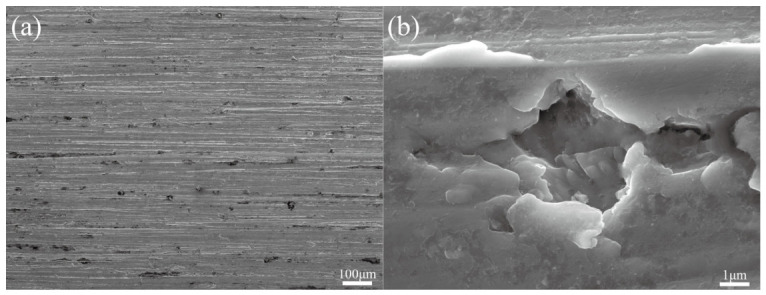
Surface morphology of TC25 titanium alloy after 10 pre-corrosion cycles: (**a**) overall view of corrosion; (**b**) corrosion details.

Figure 11 shows the corroded surface morphology of TC25 titanium alloy. As can be seen, severe corrosion damage occurred on the material surface. The surface became markedly roughened and exhibited a large number of unevenly distributed spot-like corrosion pits. These pits can induce significant stress concentration, reduce the threshold for crack initiation, deteriorate surface integrity, and thereby facilitate the initiation of fatigue cracks [51,52,53].

**Figure 12:**
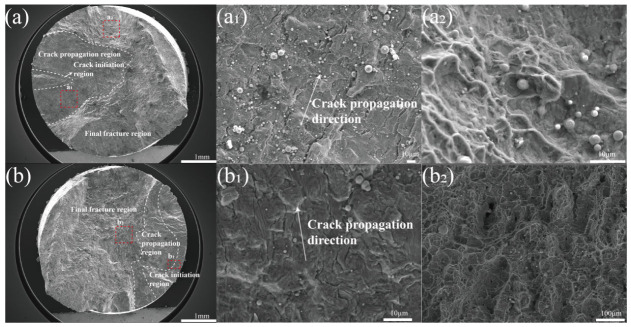
Fatigue fracture morphologies of TC25 titanium alloy before and after corrosion: (**a**) 1.0% strain amplitude before corrosion, (**a_1_**,**a_2_**) are the magnified areas of the red box in (**a**); (**b**) 1.0% strain amplitude after corrosion, (**b_1_**,**b_2_**) are the magnified areas of the red box in (**b**).

Figure 12a,b show the fracture morphologies of TC25 titanium alloy tested at a strain amplitude of 1.0% before and after corrosion, respectively. After corrosion, the number of crack initiation sites increased significantly, which can be attributed to the stress concentration caused by corrosion pits. These pits facilitate fatigue crack initiation, making the specimen more prone to premature failure during fatigue loading. Meanwhile, the rapid fracture zone expanded, whereas the crack initiation zone decreased, indicating that corrosion accelerates both crack initiation and crack propagation, thereby significantly reducing fatigue life. Figure 12(a_1_,b_1_) shows the crack propagation regions before and after corrosion, respectively. The larger striation spacing after corrosion indicates a higher crack propagation rate within the same loading cycle, which promotes faster fatigue failure of the material [54]. In addition, both Figure 12(a_2_,b_2_) exhibit obvious intergranular fracture features, suggesting that corrosion does not change the fundamental microscopic mechanism of fatigue damage.

**Figure 13:**
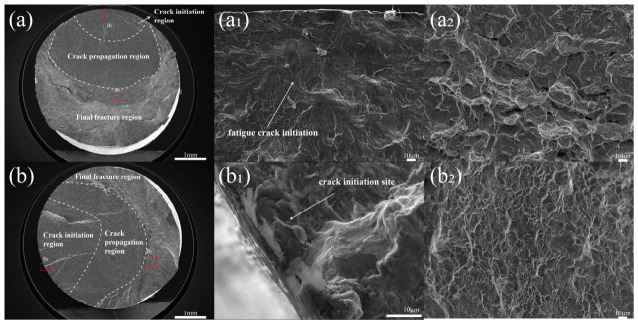
Fatigue fracture morphologies of TC25 titanium alloy before and after corrosion: (**a**) 0.6% strain amplitude before corrosion, (**a_1_**,**a_2_**) are the magnified areas of the red box in (**a**); (**b**) 0.6% strain amplitude after corrosion, (**b_1_**,**b_2_**) are the magnified areas of the red box in (**b**).

Figure 13a,b show the fracture morphologies of TC25 titanium alloy tested at a strain amplitude of 0.6% before and after corrosion, respectively. At the relatively low strain amplitude of 0.6%, the overall cyclic plastic deformation of TC25 alloy was limited, as shown in Figure 13(a_2_,b_2_), and dislocation activity in the matrix had not yet become the dominant factor controlling the fatigue behavior [22,55]. As shown in Figure 13(a_1_), before corrosion, fatigue crack initiation at a strain amplitude of 0.6% was closely associated with the α-phase region, where local strain concentration was more likely to occur during cyclic loading. After corrosion, at the same strain amplitude of 0.6%, the number of crack initiation sites increased, and crack initiation occurred from the material surface. In Figure 13(b_1_), a corrosion layer can be observed at the crack initiation site after corrosion, demonstrating that corrosion pits introduced by pre-corrosion act as local stress concentrators. During cyclic loading, these defects promote local plastic slip and dislocation pile-up [22], thereby causing earlier crack initiation and accelerating the drop in peak stress. As a result, the softening rate increased significantly after corrosion. When the strain amplitude increased to 0.7%, 1.0%, and 1.2%, cyclic plastic deformation became markedly more pronounced. Compared with Figure 13(a_2_,b_2_), more evident tearing ridges and deeper dimples can be observed in Figure 12(a_2_,b_2_), indicating that dislocation slip, dislocation rearrangement, and the accumulation of plastic strain became the principal factors governing the fatigue response [39]. Under these conditions, even the uncorroded specimens exhibited pronounced cyclic softening. Therefore, the local damage caused by corrosion defects became less significant relative to the overall plastic damage, and the difference in softening rate before and after corrosion was correspondingly reduced.

This study primarily investigated the high-temperature low-cycle fatigue (LCF) behavior of TC25 titanium alloy before and after corrosion in a simulated marine environment, including internal stress, hysteresis loops, softening degree, stress–life response curves, fatigue life equations, and microscopic mechanisms of fatigue fracture. The results indicate that marine atmospheric corrosion has a minor impact on TC25 alloy under high-strain control, whereas at low strain levels, it significantly accelerates material softening and reduces fatigue life. This phenomenon is closely related to the corrosion pits induced by marine atmospheric corrosion. This conclusion is also consistent with the findings of A. Martin et al. [12] regarding the post-corrosion high-temperature LCF behavior of single-crystal nickel-based superalloys.

## 4. Conclusions

The high-temperature low-cycle fatigue behavior of heat-treated TC25 titanium alloy after corrosion was investigated in this study. The main conclusions are summarized as follows:

1. At low strain amplitudes (e.g., 0.6%), the corroded specimens exhibited a higher cyclic softening rate compared to the uncorroded specimens. Conversely, at high strain amplitudes, this difference in fatigue behavior was less significant. We propose that fatigue failure at low strains is primarily dominated by local damage induced by corrosion defects. These defects likely act as localized stress concentrators, promoting dislocation accumulation and material degradation.
2. The cyclic softening and internal stress evolution of TC25 alloy are strongly dependent on the strain amplitude and pre-corrosion conditions. Macroscopic mechanical data indicate that at low strain amplitudes, pre-corrosion leads to an increase in friction stress and an elevation in back stress. Conversely, at a high strain amplitude of 1.2%, a different trend was recorded: the friction stress experienced a sharp drop (<100 MPa), while the back stress exhibited a significant surge (approximately 600 MPa). From a microstructural perspective, we hypothesize that corrosion defects initially act as short-range barriers at low strains. However, under high-strain conditions, severe plastic deformation is highly likely to dismantle these localized barriers, resulting in a rapid decrease in friction stress. Simultaneously, large-scale dislocation pile-ups are considered responsible for the significant increase in back stress. This critical superposition of local softening and extreme long-range stress concentration is considered the primary mechanism for the premature fracture of the fatigue specimens.
3. Fractographic analysis revealed distinct strain-dependent morphological features. At low strain amplitudes, the corroded specimens exhibited more severe surface damage and more pronounced brittle fracture characteristics. At higher strain amplitudes, both corroded and uncorroded specimens exhibited ductile fracture characteristics, including evident tearing ridges and deep dimples. These results indicate that at low strain levels, corrosion pits lead to crack initiation and reduce fatigue life, whereas at higher strain levels, plastic deformation-induced damage becomes the dominant factor.

In summary, heat-treated TC25 titanium alloy retained acceptable high-temperature low-cycle fatigue performance at higher strain amplitudes even after corrosion, whereas its fatigue resistance was markedly reduced at low strain amplitudes due to the presence of corrosion defects. These findings provide valuable insights into the application of TC25 titanium alloy in marine atmospheric and high-temperature environments. However, there are still some limitations in the present study. First, the microstructural evolution of the material at low strain amplitudes has not been comprehensively investigated. Second, as previously mentioned, the pre-corrosion treatment in this study was based on an accelerated environmental spectrum, which cannot fully capture the complex, long-term synergistic effects in actual marine service. Therefore, future work will further explore the high-cycle fatigue (HCF) behavior of TC25 alloy, incorporating multi-environmental factors and advanced microstructural characterization to bridge the gap between simulated testing and actual marine applications.

## Figures and Tables

**Table 1:** Chemical composition of TC25 alloy (wt.%) [26].

Ti	Al	W	Zr	Mo	Sn	Si
Bal.	6.48	0.72	1.02	1.78	1.12	0.13

## Data Availability

The data presented in this study are available from the corresponding author upon reasonable request, as this work constitutes part of an ongoing research project.
